# Photosynthesizing carbonate/nitrate into *Chlorococcum humicola* biomass for biodiesel and *Bacillus coagulans*-based biohydrogen production

**DOI:** 10.1186/s12934-024-02511-0

**Published:** 2024-09-11

**Authors:** Eman S. E. Aldaby, Amal W. Danial, R. Abdel-Basset

**Affiliations:** Botany and Microbiology Department, Faculty of Science, Assiut University, Assiut, Egypt

**Keywords:** Algal cake, *Bacillus coagulans*, Biohydrogen production, *Chlorococcum humicola*, C/N ratio, Fatty acids

## Abstract

Biofuel can be generated by different organisms using various substrates. The green alga *Chlorococcum humicola* OQ934050 exhibited the capability to photosynthesize carbonate carbon, maybe via the activity of carbonic anhydrase enzymes. The optimum treatment is C:N ratio of 1:1 (0.2 mmoles sodium carbonate and 0.2 mmoles sodium nitrate) as it induced the highest dry mass (more than 0.5 mg.mL^−1^). At this combination, biomass were about 0.2 mg/mL^−1^ carbohydrates, 0.085 mg/mL^−1^ proteins, and 0.16 mg/mL^−1^ oil of this dry weight. The C/N ratios of 1:1 or 10:1 induced up to 30% of the *Chlorococcum humicola* dry mass as oils. Growth and dry matter content were hindered at 50:1 C/N and oil content was reduced as a result. The fatty acid profile was strongly altered by the applied C.N ratios. The defatted leftovers of the grown alga, after oil extraction, were fermented by a newly isolated heterotrophic bacterium, identified as *Bacillus coagulans* OQ053202, to evolve hydrogen content as gas. The highest cumulative hydrogen production and reducing sugar (70 ml H_2_/g biomass and 0.128 mg/ml; respectively) were found at the C/N ratio of 10:1 with the highest hydrogen evolution efficiency (HEE) of 22.8 ml H_2_/ mg reducing sugar. The optimum treatment applied to the *Chlorococcum humicola* is C:N ratio of 1:1 for the highest dry mass, up to 30% dry mass as oils. Some fatty acids were induced while others disappeared, depending on the C/N ratios. The highest cumulative hydrogen production and reducing sugar were found at the C/N ratio of 10:1.

## Introduction

Industrialization and population growth have led to an increase in the world's energy demand. Because of rising demand, fossil fuels, which are the world's primary energy source, are becoming scarcer quickly [69]. Repeated energy crises urge continual search for sustainable fuel assets alternative to fossil fuels, especially the environmentally friendly carries such as ethanol, hydrogen and biodiesel. Microalgae are frequently pursued for their biomass conversion to sustainable biofuels because they efficiently store solar energy into biomass through photosynthesis. The ability of several microalgae, including some *Chlorella* species [37, 45, 100, 101], *Dunaliella* species [86]**,** *Nannochloris* sp. [87], *Parietochloris incisa*, *Botryococcus braunii* [44, 55], and *Coccomyxa* [51, 63] and *Nannochloris* and *Dunaliella* to accumulate significant amounts of lipids in cells under favorable conditions. Some microalgae can store up to 77% of their dry cell weight in lipids [91, 92]. Moreover, stressed microalgae produce 20–50% more lipids in their dry cell mass than they would under ideal development conditions (5–20%). The molecular formula for microalgae is CO_0.48_H_1.83_N_0.11_P_0.01_ [39] i.e., hydrogen content is almost four times as much as oxygen, giving algae an advantage over feedstocks that store carbohydrates that have a H_2_/O_2_ ratio of 2/1. Stressed algal strains, which would approach optimum lipid synthesis [39, 80]**,** have been designated as third generation feedstock [10, 20]**.** Algal biomass would also be more affordable and sustainable than other lignocelluloses or agro-industrial wastes, albeit this is vulnerable to a number of issues such as harvesting, dewatering, pretreatment expenses and the probable open-door contamination. In addition to increasing oil content, readily available nutrients also cause notable changes in fatty acid profiles. For instance, in comparison to control cultures of *Auxenochlorella pyrenoidosa*, oleic acid was observed to rise up to 60 times during nitrogen shortage [63, 104].

Algal biomass, like other organic wastes rich in carbohydrates, proteins, and lipids, can be used by bacteria that produce hydrogen gas to provide an environmentally benign, sustainable carbon–neutral fuel. An energy-efficient approach is the synthesis of bacterial hydrogen through anaerobic dark fermentation of algal biomass [75, 76, 78, 98]. To produce biohydrogen from algal biomass, bacteria including *Enterobacter* spp. [71], *Bacillus* spp. [89], and purple non-sulfur bacteria [1, 18, 19] are frequently utilized. It has been shown that a *Brevibacillus invocatus* bacterial strain may generate large yields of hydrogen using acid-hydrolyzed cyanobacterial biomass as an inexpensive carbon feedstock [27, 103]. By denying nitrogen and sulphur to *Chlamydomonas* cultures, starch enrichment [65] may encourage H_2_ generation [24]. A newly discovered strain of *Rhodobacter gokarnense* fermented yeast-hydrolyzed, nitrogen/phosphorus-deficient *Chlorella* sp. for their hydrogen content [1].

Algae need adequate supply of dissolved inorganic carbon, with CO_2_ serving as the main carbon form, for the photosynthetic carbon fixation process as well as the production of new cellular components and storage lipids. Several studies examined how atmospheric CO_2_ affected the generation of lipids or the biochemical makeup of microalgae [3, 9, 12, 21, 61, 96, 99]. Many microalgae and cyanobacteria can also actively transfer HCO_3_^−^ from the environment into the cytosol, where carbonic anhydrase produces CO_2_ in a steady-state flux to ribulose-1,5-bisphosphate carboxylase oxygenase for photosynthesis. As the pH rises above 7.0, the concentration of HCO_3_^−^ takes over, while CO_2_ takes over at a pH of less than 6.5. [11]. A variety of microalgal species have been studied using sodium bicarbonate as a carbon source for growth and biochemical composition [30, 31, 38, 66, 83, 102]. Moreover, triacylglycerol accumulation in microalgae has been demonstrated to be stimulated by bicarbonate (Yeh et al., 2012). With varying carbon circumstances, lipid accumulation in the generation of microalgal biomass and biodiesel was variable (types, concentrations, and addition methods). Carbon concentrating mechanism (CCM), Warburg's effect (the role of bicarbonate in maintaining PSII activity), and CO_2_ fixation into carbohydrates (Calvin-Benson cycle) are the three methods via which inorganic carbon participates in oxygenic photosynthesis [28]. In this study, carbonate is added as a substrate for the photosynthetic fixation of carbon. In addition to bicarbonate, carbonate has also been utilized as a carbon source for algal hydrocarbons, though much less commonly due to its greater stability and lower solubility. Carbonate is a crucial carbon source for the environment that is abundantly present in water bodies as MICP “microbially induced carbonate precipitation” [74] and in calcareous soils [70]. Subsequently, carbonate may provide a CO_2_ resource for photosynthesis, photosynthetic products, and fuels like hydrogen and oils. Carbon, followed by nitrogen are the most abundant element in microalgae biomass. The biochemical composition of algae is greatly dependent on carbon/ nitrogen ratios [8, 50, 59].

Li et al. [42] investigated the growth of co-cultivating *Chlorella vulgaris* and aerobic bacteria in mixed wastewater with different carbon to nitrogen ratios (5:1, 10:1, 15:1, 20:1, and 25:1). The carbon to nitrogen ratio has a major effect on the growth. Low carbon to nitrogen ratios encouraged the growth of *Chlorella*, but high carbon to nitrogen ratios were more advantageous for the growth of aerobic bacteria [42].

As the distribution of reducing equivalents and carbon between carbohydrates and oils in relation to nitrogenous components depends on the carbon/nitrogen ratio, the purpose of the current investigation was to assess the production of biofuels (oil and hydrogen) from the algal biomass of *Chlorococcum humicola* (chlorophyte) cultivated at varied carbonate/nitrate (C/N) ratios. A newly identified strain of *Bacillus coagulans* was mediating the fermentation of the defatted algal biomass into hydrogen. The changes that took place in the algal medium indicate accompanying the addition of the imposed treatments (carbonate and nitrate) are multiple and include pH, Electrical conductivity, salinity, Ca^*2*+^and Mg^*2*^, which singly or collectively are physiological factors that affect cellular metabolism and hence their growth.

## Materials and methods

### Experimental set up

The Chlorophyte *Chlrococcum* (*Chl.*) *humicola* was isolated from Al-Elibrhmia canal (Assiut, Egypt), and enriched in 250 mL Erlenmeyer flasks containing the liquid algal nutritive medium BG11 [84], starting from a fixed optical density (0.03 A). Various C/N ratios, composed by additional carbonate to BG11 medium, are shown in Table 1 as follows:

**Table 1:** Different treatments of C/N ratios (1:1, 10:1, 30:1 & 50:1) in comparison with control (BG11) or deprived of nitrogen 1:0(BG11−), as follows

Nutrients (mmole/L)	(BG11) control	(BG11−) 1:0	Carbonate/nitrate ratios			
			1:1	10:1	30:1	50:1
Na_2_CO_3_	1.886	1.886	0.2	2	6	10
NaNO_3_	0.176	0	0.2	0.2	0.2	0.2

The treated cultures were grown for two weeks, at 100 µmol photons m^−2^s^−1^ 28±2 ºϹ under continuous white light and 100 rpm agitation using a MiniOrbital Shaker (VWR, USA). The effect of the above ratios was followed on growth parameters, oil content and hydrogen evolution of the algae.

The applied treatments of Na_2_CO_3_ and NaNO_3_ were accompanied with obvious changes in the physical and chemical characteristics medium.

### Analytical methods

The algal cultures were harvested by centrifugation, and the cells were then washed in sterilized distilled water to eliminate any remaining growth media. Optical density of the alga (OD750 nm) was determined according to Zhao et al [106]. For the determination of dry mass, algal suspensions (20 mL) were filtered through a glass fiber filter. Then, the filter papers with algal cells were dried overnight in an oven at 80 ºϹ. After cooling, they were weighed, and the dry mass was calculated as mg/mL. Photosynthetic pigments (total chlorophyll and carotenoid concentrations) were extracted for 10 minutes in hot methanol, centrifuged and the supernatant was measured spectrophotometrically at 663 nm, 644 nm and 452 nm and pigment contents were calculated as (μg/mL) using Marker's method [53]. Soluble carbohydrates (μg/mL) in the supernatants were estimated by the anthrone-sulphuric acid method as previously described [25, 73]. Soluble proteins were determined as μg/mL according to the method of Lowery et al [48]. Lipid extraction and determination in 200 mL aliquots of each algal culture; they were centrifuged at 5000 rpm for 15 min. and the pellet was homogenized in 4 mL of ice-chilled chloroform: methanol mixture (2:1 v/v) in Falcon tubes according to Hara and Radin [33] and gently shaken to extract the lipids from the cells. About 4 mL ice-chilled 1M MgCl_2_ solution were added to the above mixture to sharply separate the two liquid phases. The lower (chloroform) phase contains the algal lipids and is drawn off from the tube *via* a long syringe needle, which is then transferred into dry pre-weighed aluminum foil cups, evaporated, and then reweighed. Lipids were assessed as oil content (per unit culture) and oil percentage (relative to unit dry mass), as oil content was calculated as mg/mL algal culture and oil percentage.

$$\text{Oil percentage }= \frac{\text{mg oil}}{\text{mg dry mass}} x 100$$

### Determination of fatty acid profile

Fatty acid profiles of the different oil extracts were esterified using methanol/sulfuric acid mixture. Thereafter, fatty acid methyl esters (FAMEs) were quantified using the GC mass (Shimadzu, GC-MS 2014) equipped with a thermal conductivity detector (TCD) and Shin Carbon packed column (ST 80/100 2 m, 2 mm ID). Argon was used as carrier gas.

### Bacterial isolation and identification

A local isolate of heterotrophic bacteria (B1) was isolated from soil in Assiut University, Assiut, Egypt and grown anaerobically in nutrient broth/agar medium at 30 °C. Colonies were analyzed macroscopically considering colony color, length and width. The colony size and shape were determined using light microscopy and the genotypic characterizations of the isolate were assessed.

### Phylogenetic analysis of *bacteria*

The morphological and molecular properties were used for strain identification according to Brenner et al. [7]**.** Based on 16S rRNA, bacterial isolate was genetically identified. Following the manufacturer's instructions, total genomic DNA was extracted and purified from the isolated species using the GeneJet Genomic DNA purification Mini Kit (Thermo Scientific). The amplification was conducted using a CreamCon thermal cycler (Holand). As instructed by the manufacturer, Green Taq (Dream Taq) master mix (Thermo Scientific) was used for gene amplification. Using the universal forward primers 27F (5′-AGAGTTTGATCCTGGCTCAG- 3′) and 1292R (5′- CTACGGCTACCTTGTTACGA- 3′), the 16S rRNA region of bacteria was amplified. The thermal cycler conditions began with a 3-min denaturation at 94 °C, then 35 incubation cycle with 1 min denaturation at 95 °C, 1.5-min annealing at 95 °C, 2-min elongation at 72 °C, and a final 5-min denaturation at 94 °C. Gene JET PCR Purification Kit was used to purify the PCR results (Termo Scientifc). Macrogen In. seal, Korea, applied the ABI PRISM® 3100 Genetic Analyzer for PCR products. The purified products were sequenced and aligned with various 16S rRNA through the nBLAST search (http://www.ncbi.nih.gov/blast) to find the closely related species using MEGA 11's default Clustal W parameters. Additionally, a dendrogram based on the neighbor-joining (NJ) technique and the parameter distance (PD) was created using the MEGA 11 software [56].

### Molecular identification of algae

The microalgal culture was centrifuged and the pellet was utilized for DNA extraction. One milliliter of culture was sonicated in a water bath for 10 min. DNA was extracted using a DNA easy Plant Mini Kit (Qiagen, Hilden, North Rhine-Westphalia, Germany) according to the manufacturer's instructions. Extracted microalgal DNA was subjected to agarose gel electrophoresis using 1.0 × TAE buffer (44.5  mM Tris, 44.5  mM glacial acetic acid and 1.0  mM EDTA), followed by staining with GelRed solution (Biotium, Fermont, Quebec, Canada) and visualization under UV light. The purity of the extracted microalgal DNA was assessed in terms of absorbance ratios at 260 nm/230 nm and 260 nm/280 nm. PCR amplification and sequencing of the 18S rRNA gene Oligonucleotide primers from published studies and designed primers were utilized in the present study to amplify the 18S rRNA gene of the tested microalgae (ss5 (F) GGTGATCCTGCCAGTAGTCATATGCTTG and ss3 (R) GATCCTTCCGCAGGTTCACCTACGGAAACC). PCR was performed in a total reaction volume of 25 μL containing 1.0 × PCR buffer, 3 mM MgCl_2_, 0.2 mM dNTP (Fermentas, Waltham, Massachusetts, USA), 1.0 μM of each forward and reverse primer, 2.5 U of Pfu DNA Polymerase (Promega, Madison, Wisconsin, USA) and 3 μL of template. The PCR protocol comprises an initial denaturation step at 95 °C for 5 min, followed by 35 cycles of 95 °C for 30 s (denaturation step), 50 °C for 1 min (annealing step) and 72 °C for 1 min (extension), and final extension at 72 °C for 7 min with the use of a Tpersonal Thermal Cycler (Biometra, Gottingen, Lower Saxony, Germany). The PCR products were then gel-purified using a gel/PCR DNA fragment extraction kit (Geneaid, New Taipei City, Shijr District, Taiwan).

The sequence was aligned with various 18S rRNA, treated as shown above for bacteria. to find the closely related species and fit into a dendrogram based on the neighbor-joining (NJ) technique.

### Nucleotide sequence accession numbers

The nucleotide sequence of the bacterial isolate B1 (*Bacillus coagulans*) and algal isolate (*Chlorococcum humicola*) were deposited in the database of GenBank nucleotide sequence under accession number of OQ053202 and OQ934050; respectively and the Phylogenetic tree including the strains was illustrated.

### Biohydrogen production

The bacterial isolate was grown for 24 h in minimal medium (MM), prepared by dissolving 1.0 g Na_2_HPO_4_, 0.2 g of KCl and 0.2 g of MgSO_4_ in 1 L of distilled water [56]. Bottles containing hydrogen evolving cocktails (10% phosphate buffer pH 6.8 ± 0.2,10% early log phase bacteria i.e., 24 h old cells from MM, 80% algal biomass suspended in MM) to obtain a total volume of 1 L; bottles were stoppered and flushed with nitrogen for 15 min. They were then kept at 30 °C and stirred by magnetic stirrer as long as hydrogen was evolving i.e., three days. The evolved biohydrogen gas was determined by gas chromatograph apparatus (Thermo Scientifc TRACE GC Ultra) equipped with a thermal conductivity detector (TCD) and Shin Carbon packed column (ST 80/100 2 m, 2 mm ID). Argon was used as carrier gas. The temperature at the column was initially 50 °C and was increased to 250 °C at a rate of 30 °C/min.

For each treatment, triplicates were performed, and the average values were taken as the final result. A control test was performed using a fermentative medium containing glucose. An additional blank assay with only an anaerobic medium without any substrate or inoculum also acted as a negative control.

### Calculation of hydrogen evolution efficiency (HEE)

Conversion of biomass into hydrogen gas evolved was calculated once as the hydrogen evolution efficiency per unit dry mass (mlH_2_. g^-1^ dry mass) and second as the hydrogen evolution efficiency per unit of consumed reducing sugars (mlH_2_. mg^-1^ reducing sugars).

### Statistical analysis

Each experiment was repeated three times and the mean value of three replicates ± standard error (SE) is presented. Statistical analysis of the data was conducted using ANOVA one-way test (analysis of variance) by SPSS program version 21, and Duncan values were determined at 0.05 level. Different letters (a-f) on the graphs and tables indicate significant differences between the treatments.

## Results

### Identification of algal culture

**Fig. 1:**
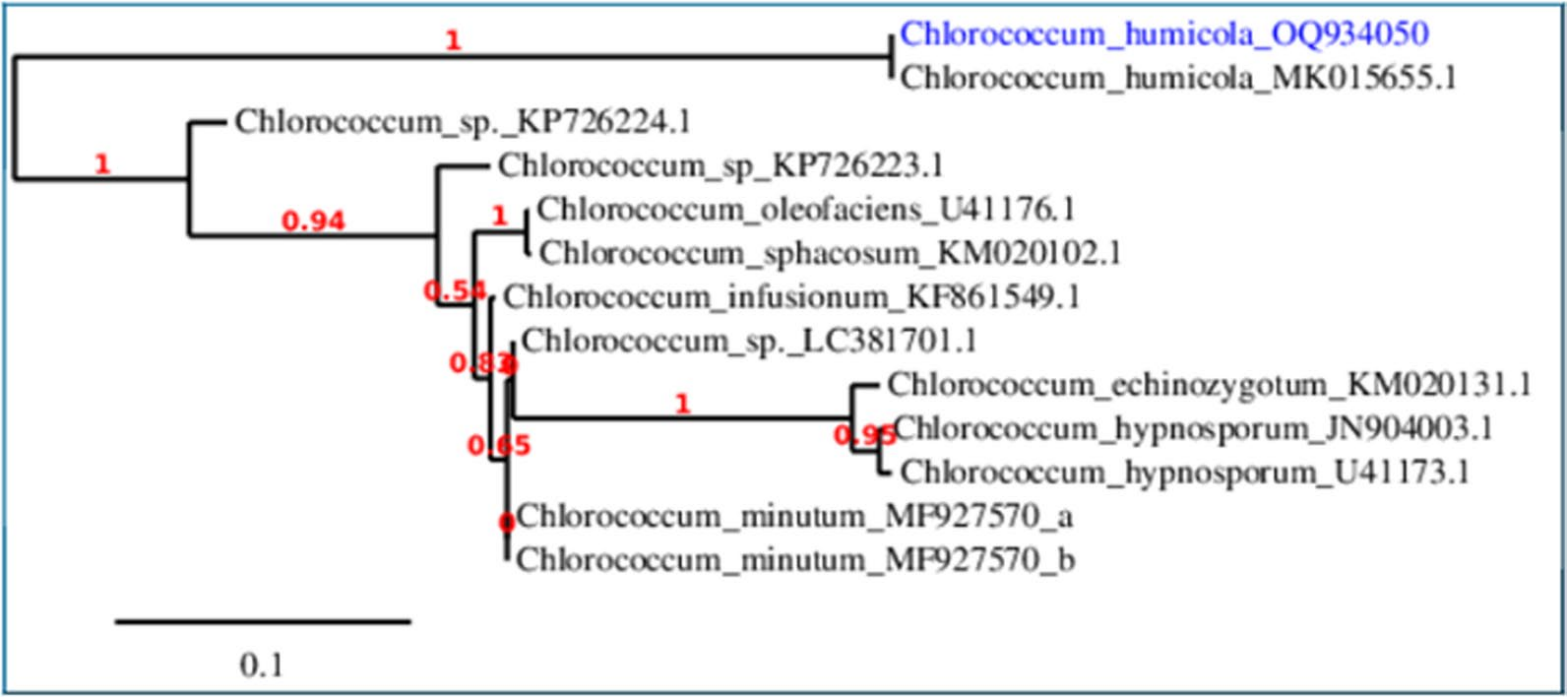
Phylogenetic tree on the basis of patterns and genetic relationship of *Chlorococcum humicola*

In this work, *Chlorococcum humicola* OQ934050 was identified genetically (Fig. 1) and revealed its ability to utilize carbonate as a CO_2_ resource for photosynthesis and thus photosynthetic products, biofuels (oils and hydrogen-bearing biomass in this work).

### Some properties of the growth medium

Table 2 shows the physical and chemical characteristics of the basal medium as a result of carbonate/nitrate additions. These are the elevated pH (from 7.2 to 10.0), elevated EC (from 1171 to 1973 µS.cm) and severely decreased Ca^*2*+^.

**Table 2:** Some properties of the growth medium as influenced by the imposed treatments

Treatments	pH	EC ( µS.cm)	Ca^2+^ ( mg/)	Mg^2+^ ( mg/L)
Control	7.2	1171	69.2	17.41
C/N (1/0)	7.3	1141	70.8	17.28
C/N (1/1)	8.7	1140	70.7	16.47
C/N (10/1)	9.5	1205	75.3	18.23
C/N (30/1)	9.9	1366	35.1	15.61
C/N (50/1)	10.0	1973	16.6	14.97

### Effect of C/N ratios on the growth parameters

**Fig. 2:**
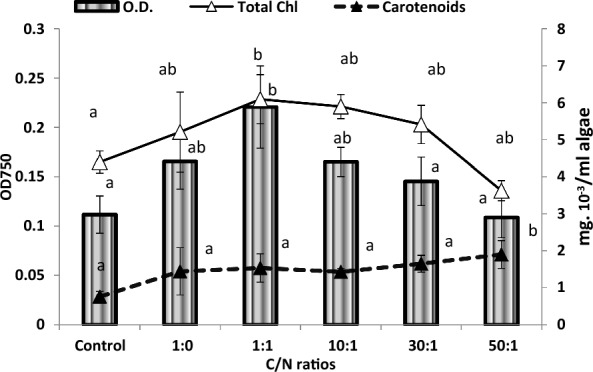
Optical density, chlorophyll content and carotenoids content of *Chlorococcum humicola* under the effect of various C/N ratios (1:1, 10:1, 30:1 & 50:1), as compared with control (BG11) and nitrate deprived cultures 1:0 (BG11−)

This study dealt with the influence of successively increasing carbonate/nitrate ratios of 1:0, 1:1, 10:1, 30:1 & 50:1 (mM/mM sodium salts), with the nitrogen content being constant as 0.2 mM NaNO_3_, in addition to control (BG11) and nitrogen deprived (BG11−) cultures. Growth, biochemical composition, oil content and hydrogen production of the green alga *Chlorococcum humicola* were followed. Augmented carbon/nitrogen ratios enhanced optical density (O.D.750) of *Chlorococcum humicola* up to the ratio of 30:1, compared with the control culture (Fig. 2). The highest enhancement of O.D.750 nm of *Chlorococcum humicola* was reported as 0.22A at 1:1 (0.2 mol L^−1^ sodium carbonate with 0.2 mol L^−1^ sodium nitrate). Similarly, total chlorophyll content of *Chlorococcum humicola* was increased from 4.4 × 10^–3^ mg/ mL at control culture to 6.0 × 10^–3^ mg/ mL at C/N ratio of 1:1 (Fig. 2), whereas the lowest content of chlorophyll was recorded at the highet ratio of 50:1 (i.e., at 10 mol L^−1^ of sodium carbonate with 0.2 mol L^−1^ sodium nitrate). Results also show that nitrogen deprivation has no negative impact on chlorophyll content in comparison with the control but, otherwise, it was relatively increased. All treatments enhanced the carotenoid contents in *Chlorococcum humicola*; in comparison with the control; increasing the C/N ratio increased carotenoids content up to almost threefold at 50:1 ratio (Fig. 2); furthermore, nitrogen deficiency raised carotenoids content by almost 90% relative to the control.

**Fig. 3:**
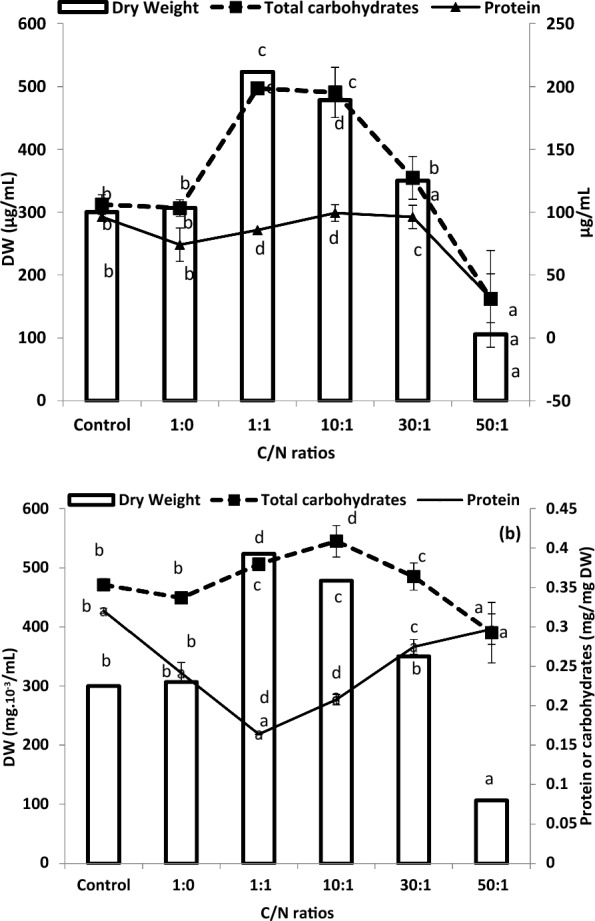
Dry weight, total carbohydrates and protein content of *Chlorococcum humicola* grown as shown in Fig. 2

Growth (dry mass/ml algal culture) was increased by almost all C:N ratios up to 30:1 when compared with the control or with the highest ratio of 50:1. The maximum dry weight was found at the ratio of 1:1 C/N, as 75% higher than the control (Fig. 3); maximum dry mass indicates that such carbonate/nitrate ratio of 1:1, at the concentration of 0.2 mol sodium carbonate with 0.2 mol sodium nitrate, is the optimum ratio for growth and it is the most balanced C/N ratio. This ratio simultaneously maximized carbohydrate contents to which the dry mass of *Chlorococcum humicola* while protein content was somewhat reduced (Fig. 3). However, the data revealed that the increase in Na_2_CO_3_ has a detrimental decreasing effect on growth and chemical composition of *Chlorococcum* when it is not accompanied by an increase in NaNO_3_. Carbohydrates decreased at C:N ratio of 30:1. The ratio of 50:1 (10 mol L^−1^ with 0.2 mol L^−1^ sodium nitrate) sharply decreased dry mass, carbohydrates and proteins per unit volume (/ml algal suspension), in concomitance with theremarkably less growth (e.g. O.D.750 nm). However, per unit dry mass (Fig. 3), protein content, however, was noticeably increased at C/N ratio of 50:1, due to the higher cellular quotas of available nitrogen (less cell number) relative to carbon. However, the presence or absence of nitrogen (in BG11 or BG11−) has no effect on the amount of carbohydrates present and dry mass. Figure. 3 shows that depletion of nitrogen (C:N ratio of 1:0) has a direct negative impact on protein content; as nitrogen is a major component of amino acids; its deficiency reduced protein levels by nearly 23% compared with the control culture. Similarly, the amplified carbonate/nitrate ratio of 50:1 severely inhibited protein content to about 30% that of the control; it may be also due to the reduction of dry matter of *Chlorococcum* under this ratio (Fig. 3). At 10:1 and 30:1 C:N ratios, protein content elevated again to be almost equal to the control.

In summary, it can be concluded that the optimum treatment is C/N ratio of 1:1 (0.2 mM sodium carbonate and 0.2 mM sodium nitrate); as this ratio resulted in the highest dry mass (more than 0.5 mg/mL^−1^), about 0.2 mg/mL^−1^ carbohydrates but not proteins (0.085 mg/mL^−1^ proteins). At 1:1 and 10:1 C/N ratios, up to 30% of the dry weight of *Chlorococcum humicola* can be utilized in oil production (0.160 mg/ mL^−1^). Other ratios resulted in markedly less oil percentage ranging from 15 to 20% of the dry mass while extremely low oil percentage (only 1%) was recorded at C/N ratio of 50/1.

### Effect of C/N ratios on oil content and yield

**Fig. 4:**
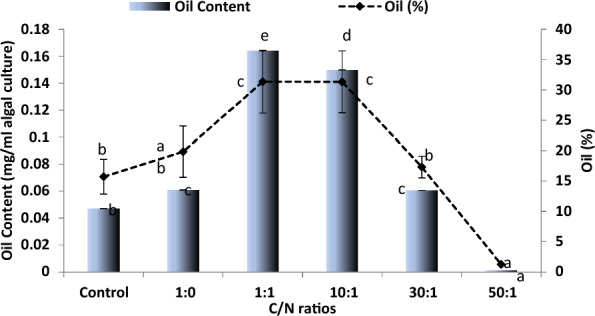
Oil content (mg/ ml algal culture) and oil percentage (relative to dry mass) of *Chlorococcum humicola* grown as shown in Fig. 2

Oil content (mg/malgal culture) and oil percentage (relative to the dry mass) of *Chlorococcum* were boosted at 1:1 and 10:1 C/N ratios (Fig. 4). The content increased by almost four-fold that of the control culture while the percentage spiked to over 31% at the C: N ratio of 10:1, compared to roughly 15% oil percentage in the control of *Chlorococcum*. Nitrogen deprivation (C/N of 1:0) relatively enhanced oil content and percentage; the less nitrogen the higher carbohydrates/ oils accumulate, when nitrogen is limited. However, in comparison with the control, the C/N ratio of 30/1 resulted in a slight increase in oil content and percentage (27 and 10, respectively).

Furthermore, major oil contents at certain treatments (Control, C/N 1/0, 1/1 and 10/1) were methylated and analyzed for fatty acid methyl esters (FAMEs) using GC mass spectrometry; the resulted FAMEs are presented in Table 3. The fatty acid profiles (FAs) were noticeably altered. Control cultures of *Chlorococcum humicola* displayed only three FAMEs; they are oleic acid, eicosyl esters, docosanoic acid, 1,2,3-propanetriyl ester and pentadecanoic acid, 13-methyl-, methyl esters at percentages of 27.9, 62.7 and 9.4%, respectively, relative to the total FAMEs detected (Table 3). Nitrogen deprivation (C: N of 1:0) at BG11− induced three more FAMEs; these are linoleic acid ethyl esters, hexadecanoic acid, methyl ester and decanoic acid esters of 1.6, 6.4 and 1.6%, respectively. The C:N ratio of 1:1, which is the optimum for growth, dry mass and oil content was accompanied with only four FAMEs; with eicosatetraynoic acid, methyl esters and docosatetraenoic acid ester being first induced among all the treatments whereas pentadecanoic acid, 13-methyl-, methyl esters and decanoic acid esters disappeared. The C:N ratio of 10:1 induced six FAMEs with two newly induced ones (dodecanoic acid, 2-phenyl-1,3-dioxan-5-yl esters and eicosanedioic acid, dimethyl esters).

**Table 3:** Fatty acid methyl esters (FAMEs) of *Chroococcum humicola* grown for 15 days at successively increasing carbonate/nitrate concentrations (Control, 1:0, 1:1, 10:1 C/N)

FAME	Abbreviation	Control	C/N 1/0	C/N 1/1	C/N 10/1
1. Linoleic acid ethyl esters	18:2	–	1.6%	2.2	4.3
2. Oleic acid, eicosyl esters	18:1	27.9%	81.9%	57.8	32.4
3. Docosatetraenoic acid ester	22:4	–	–	3	–
4. Docosanoic acid, 1,2,3-propanetriyl esters	22:0	62.7%	4.3%	13.8	33.56
5. Hexadecanoic acid, methyl ester	16:0	–	6.4%	22.7	10.1
6. Eicosatetraynoic acid, methyl esters	20:4	–	–	0.48	–
7. Pentadecanoic acid, 13-methyl-, methyl esters	15:1	9.4%	4%	–	–
8. Decanoic acid esters	14:0	–	1.6%	–	–
9. Dodecanoic acid, 2-phenyl-1,3-dioxan-5-yl esters	12:0	–	–	–	2.1
10. Eicosanedioic acid, dimethyl esters	20:0	–	–	–	0.22

After extracting oils, the hydrogen content of the remaining defatted biomass of *Chlorococcum humicola* (algal cake, rich in carbohydrates, proteins, sugars and amino acids), has been used as a substrate for hydrogen production by bacteria.

### Identification of bacterial strain

**Fig. 5:**
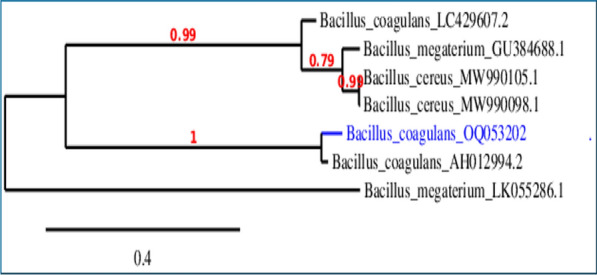
Phylogenetic tree on the basis of patterns and genetic relationship of *Bacillus coagulans*

*Bacillus coagulans* OQ053202, genetically identified using 16S rRNA (Fig. 5), which used as hydrogen producer strain.

## Hydrogen production by *bacteria*

**Fig. 6:**
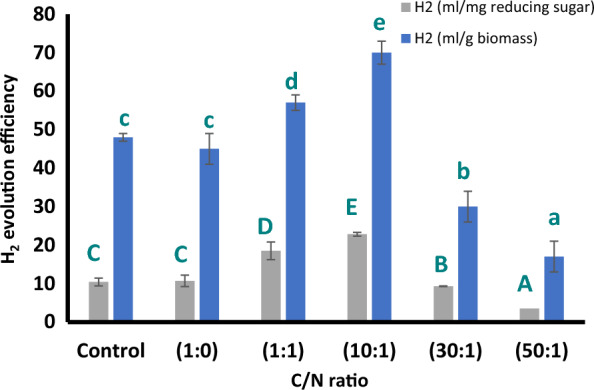
Hydrogen evolution efficiency (HEE) of *Chlorococcum humicola* biomass variously C/N ratios (Control, 1:0, 1:1, 10:1, 30:1 & 50:1) in compared with control (BG11) by *Bacillus coagulans *per unit reducing sugars and per gram biomass

The highest hydrogen evolved from *Chlorococcum* defatted cake grown at C:N ratio of 10:1, followed by C:N ratio of 1:1; control and nitrogen deprivation induced more or less similar amounts of hydrogen while the least amounts were evolved at 30:1 and 50:1 (Table 4). Hydrogen efficiency exhibited the highest rate at carbonate/nitrate ratios of 1:1 and more at 10:1; the lowest at 30:1 and 50:1; nitrogen deprived, and control cultures exhibited almost the same amount of efficiency (Fig. 6).

**Table 4:** Cumulative hydrogen of *Chlorococcum humicola* biomass variously C/N ratios (Control, 1:0, 1:1, 10:1, 30:1, 50:1) by *Bacillus coagulans*

Carbon nitrogen ratio	Control	1:0	1:1	10:1	30:1	50:1
Cumulative H_2_ ( ml/50 ml culture)	5	13	10	14	5	3

## Discussion

**Fig. 7:**
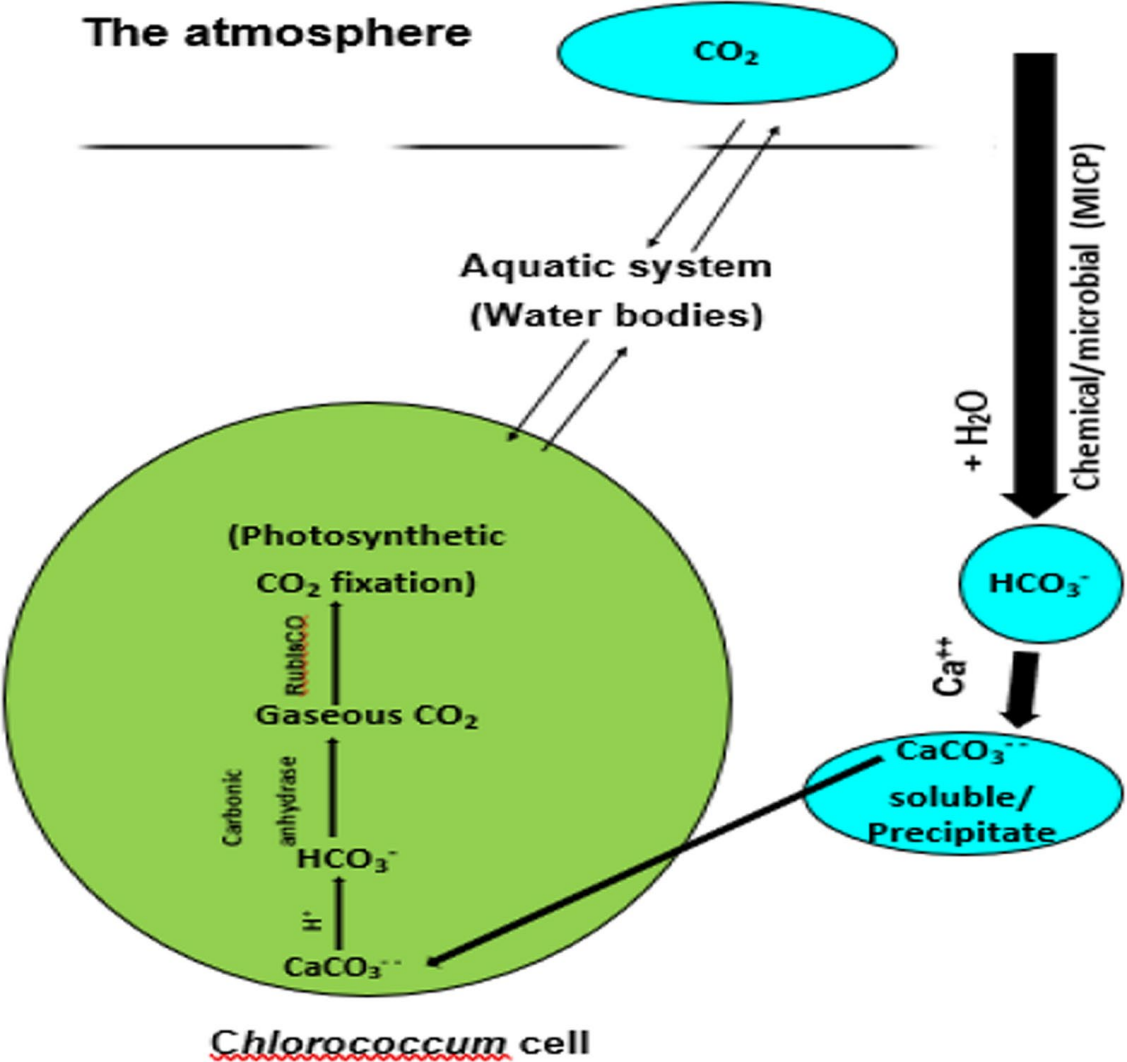
A proposed schematic representation of calcium carbonate formation from atmospheric CO_2_, in aquatic systems and its dissociation to CO_2_ via the reversible activity of carbonic anhydrase; the evolved CO_2_ is to be utilized as a photosynthetic substrate of *Chlorococcum humicola*

In photosynthesis, gaseous CO_2_, is fixed as the primary carbon form and incorporated via the enzyme ribulose-1,5-bisphosphate carboxylase/oxygenase (RubisCO) into organic compounds utilizing solar energy. In addition to CO_2_, HCO_3_^−^ is photosynthesized via the activity of the carbonic anhydrases (CA) or carbonate dehydratases (EC 4.2.1.1), which catalyzes the interconversion of HCO_3_^−^, CO_2_ and H_2_O and carbonic acid, a fundamental reaction for life. CA impacts numerous physiological processes in plant and animal body, namely acid–base as well as fluid balance and helps transport carbon dioxide. βCAs are found in chloroplast stroma, mitochondria, the cytosol, and the plasma membrane, to regulate CO_2_/ HCO_3_^−^ in photosynthesis and respiration. Indeed, sodium bicarbonate has been studied to provide adequate carbon source for intensive growth and biochemical composition in commercial microalgae production where CO_2_ sources, e.g. flumes from power stations, etc., are not readily available [30, 102]. The addition of bicarbonate also induced growth recommencement of *Ccoccomyxa chodatii* and *Rhodobium gokarnense* [19]. Furthermore, bicarbonate nutrition has been shown to stimulate triacylglycerol accumulation in microalgae [29], in the course of biofuel production from algae. Other than carbon fixation in Calvin–Benson cycle and carbon concentrating mechanism (CCM) [34], inorganic carbon affects PSII activity via Warburg’s effect reviewed in Shevela et al. [77]. In this work, sodium carbonate is introduced as the substrate for photosynthetic carbon fixation in *Chlorococcum humicola*. When dissolved in water, sodium carbonate forms carbonic acid and sodium hydroxide; carbonic acid in water gives off CO_2_, which is readily photosynthesized. Otherwise, photosynthesizing sodium carbonate by algae takes place after it is first validated by the action of carbonic anhydrase enzyme in algae and bacteria (*Chlorococcum humicola* and *Bacillus coagulans*, in this case); the dissociation products bicarbonate and CO_2_ can be photosynthesized by the chlorophyte *Chlorococcum Humicola*. carbonic anhydrase accomplished its validation to CO_2_, as inferred from growth capability and enhancement (O.D. 750 nm), chlorophyll dry mass, carbohydrates and proteins. Such observation may be attributed to the nitrogen storage in the mother cells that is enough to the divided cells throughout the short duration of the experiment (15 days). In agreement with our results, Barajas-Solano et al. [6] found that sodium carbonate enhanced the biomass and hydrocarbon production in a Colombian strain of *Botryococcus braunni*. As the C:N ratio rose, the content of chlorophyll first increased and subsequently declined [42]. In this work, *Chlorococcum humicola* efficiently photosynthesized and incorporated carbonate into algal biomass; photosynthesizing carbonate indicates environmental implications in the sense of extending the sink size of carbonate for further carbon precipitation, chemically or microbially i.e. CO_2_ may be drained from the atmosphere. When the C/N ratio was raised to 16, Visentin et al., [93] observed that the biomass composition of *Phormidium autumnale* was enhanced in terms of proteins, carbohydrates, lipids, and phycocyanin. A schematic representation of a proposed CO_2_-carbonate cycle is presented in Fig. 7, showing that carbonate plays a dual role in source/ sink for CO_2_, from which CO_2_ is photosynthesized and at which it precipitates**.** In this hypothesis, carbonate is degraded to CO_2_, photosynthetically fixed, enabling dissolution of atmospheric CO_2_ with water to form bicarbonate up to a chemical equilibrium; both reactions, degradation and formation, are catalyzed by the carbonic anhydrase activity in a *Chlorococcum* cell and its aqueous environment. A possible positive environmental impact may arise, as CO_2_ is finally drained from the atmosphere. In this context, Albert Ng and Liu [2] stated that with greater C/N ratios, carbon is abundant, nitrogen is scarce, and the rise in cell lipid content can be interpreted as a carbon storage mechanism. Duarte (1992) pointed out that differences in C content between species reflect the differences in structural C but not of C associated with metabolic processes; total C contents of the species are similar to values of macroalgae from other regions. Morales-Plasencia et al. [59] reported that nitrogen limitation (N-limitation) a crucial factor that modifies their biochemical composition. In *Nannochloropsis* cultures, N-limitation can cause lipid or carbohydrate accumulation. The 25% and 0% N treatments had a negative effect (*P* < 0.05) on cell population growth, carbohydrates, and β-glucans accumulation. Algal growth, per se, could change the pH due to producing OH^−^ while removing fixing CO_2_ and HCO_3_^−^ during photosynthesis, which changes the equilibrium and elevates the pH [59]. The condition of low C/N ratio was conducive for cells to intake sufficient nutrients from the medium for metabolism, thus promoting rapid cell division and biomass accumulation [8], particularly nitrogen for enzymes proteins build up. When the C/N ratio was risen, the biomass productivity of the *Scenedesmus* sp. *Z-4* dropped due to nutritional deficiency [50], particularly nitrogen when does not match the demand of carbon metabolism. The data, in this work, coincided with the above literature as it revealed the modification of the structural proportions of the green alga *Chlorococcum humicola* cells as consequent to C/N alterations. Furthermore, the increase in Na_2_CO_3_ has a detrimental decreasing effect on growth and chemical composition of *Chlorococcum* when it is not accompanied by an increase in NaNO_3_.

The highest C/N ratio of 50:1 was stressful as it exerted a negative impact on oil content and percentage, could be owing to poor growth as a result of the high sodium carbonate in growth media. However, the magnitude of stress on oils was more pronounced than on either dry mass, carbohydrates or proteins. In this respect, several species and strains belonging to the genus *Coccomyxa* have been intensively studied to identify the nutritional parameters for growth, which led to an increase in lipid yields [5, 51]. The results obtained in this work using *Chlorococcum humicola*, agreed with the notion that nitrogen deficiency raised carotenoids content, which are hydrocarbons, by almost 75% compared with the control. The increased carotenoids content may function as antioxidants to stabilize and protect photochemical processes of photosynthesis in a stressful environment [97], accompanied with decreased in chlorophyll [67]; carotenoids are important parts of the light harvesting antennae and also as hydrocarbons, indicate high energetic proportion in *Chlorococcum* cells. Cai et al. [8] found that the reduction of C/N ratio in the growth medium promoted the synthesis and accumulation of glutamate in microalgae, which in turn promoted the anabolism of other amino acids; collectively, further contributed to the enhancement protein synthesis and accumulation. When the C/N ratio was elevated to 12:1, the biomass productivity and protein content attained higher levels becoming 0.90 g/L/day and 61.56%, respectively. The obtained *Chlorella vulgaris* biomass was rich in essential amino acids (41.80%), the essential amino acid index was 89.07, and the lysine content could reach up to 4.05 g/100 g. Recently, Gao et al. (2023) recorded the enhancement of photosynthesis (chlorophyll a, chlorophyll b, carotenoid content, Rubisco activity, net photosynthetic rate, D1 protein via promoting psbA expression, enhanced photosynthetic carbon assimilation by increasing Rubisco activity via promoting rbcL expression transpiration rate, and stomatal conductance, growth, antioxidants and development of wheat seedlings by ordinary -or nano-calcium carbonate.

In this work, the highest biomass production and organic content of *Chlorococcum humicola* was recorded at C/N ratio of 10:1, reduced at lower or higher ratios; similar to that recorded in a symbiotic microalgae-activated sludge system treating wastewater [14]. Furthermore, they found that the co-culture of *Chlorella* sp. and activated sludge could achieve synergy impact on biomass production under the best C/N ratio. Low or high C/N ratios means insufficient inorganic carbon for photosynthesis, leading to lowered biomass production of microalgae [49, 90, 107]. In contrast, when the C/N rate has risen from 5:1 to 20:1, the biomass productivity of the *Scenedesmus* sp. *Z-4* dropped due to nutritional deficiency [50]. A proper C/N ratio operation facilitates algal metabolism and their ability to effectively absorb nutrients [57, 81, 85]. found that N fertilization increased N concentrations and decreased the C:N ratio, while the effects of fertilization on total C concentrations varied with tree species and organs. Concentrations of non-structural carbohydrates (mainly reflected in soluble sugar) were generally negatively correlated with N concentration in fine roots but positively related to N concentration in aboveground woody organs in both control and fertilized treatments. However, fertilization strengthened this correlation in fine roots and weakened this relationship in aboveground organs. Reyes et al. [68] finalized the conclusion that the tight coordination between C and N not only affects the carbohydrates metabolism and their concentration within plant tissues, but also the partitioning of the excitation energy at PSII level between radiative (electron transport) and non-radiative (heat) dissipation pathways.

The optimum treatment for growth (highest dry mass) is C/N ratio of 1:1 (0.2 mM sodium carbonate and 0.2 mM sodium nitrate), similar to 10:1 C/N ratio, were accompanied with up to 30% of the dry weight of *Chlorococcum humicola* as oils. (0.160 mg/ mL^−1^). Other ratios resulted in markedly less oil percentage ranging from 15 to 20% of the dry mass while extremely low oil percentage (only 1%) was recorded at C/N ratio of 50/1. The fatty acid profile showed that some fatty acids were induced, and some others disappeared in response to carbonate and nitrate combinations. Collectively, the type and proportion of FAMEs (fatty acid methyl esters) exhibited dependence on the C:N ratio; the number of fatty acids seems proportional with C/N ratios. Deprivation of N as well as excess C (10/1) enhanced the number of FAMs detected to the double of control cultures (6 and 3; respectively). The total FAs yield of *Coccomyxa* sp. under nutritional stress often implies low biomass yield and significant variations in FAs profiles [5, 51, 72], as reported previously in *Coccomyxa elongata* MZ-Ch64 under different nutrient deficiencies of nitrogen and phosphorus [51, 63]. Similarly, *Chlorococcum humicola* exhibited comparable attitude under the studied carbonate/nitrate combinations. Also, FAs biosynthesis can be also triggered under nutrient starvation, when nitrogen [63, 95] or phosphorus (60) is depleted from the culture medium of *Neochloris oleoabundans*, *Coccomyxa AP01* and *Chlorella ellipsoidea* (CUH/Al/MW-189) and* Chlorococcum infusionum* (CUH/Al/MW-190). For instance, in *Auxenochlorella pyrenoidosa*, oleic acid was reported to increase up to 60 times under nitrogen starvation, with respect to control cultures [63, 104], and it was suggested that nitrate could induce desaturation of C18 FAs. Also, Los and Murata [47] reported significant variation in fatty acids biosynthesis according to the nutrients supplemented. CO_2_ seems to control intracellular fatty acid composition and content [35, 64]. On the other side, nitrate concentration in media can control biosynthesis of fatty acids; triglyceride-fatty acid synthesis was promoted by starvation of nitrate in a culture media of some microalgae, *Botryococcus braunii* and *Chlorocuccum littorale* [13, 44, 64].

In addition to FAMES, extracts showed four major unsaponifiable compounds having pharmaceutical importance, phenol, 2,4-bis (1,1-dimethylethyl) is the natural product that exhibits potent toxicity against almost all testing organisms, [105]**,** gibberellic acid ester is a growth hormones, phthalic acid is a the modest commercial importance, plasticizers added to polymeric materials [36, 46], butyl tetradecyl ester and Ethyl iso-allocholate are **a**ntimicrobial, diuretic and anti-inflammatory activities [79]. In this respect, Kumar et al. [41] reported on algae as potential feedstock for the production of biofuels and value-added products.

Biohydrogen is a type of green hydrogen, in addition to electrolysis, which is still a matter of utmost importance to fulfill global energy demand in substitution of fossil fuels, with the lowest pollution level and the lowest possible cost [82, 94]. The energy content of hydrogen per unit weight is higher than any other hydrocarbon. Bacteria possess several hydrolytic enzymes can breakdown complex structure [54]. Bacteria can breakdown of algal biomass to simpler compounds (sugars, organic and amino acids), and used as a nutrient media for bacterial growth and hydrogen evolution by their nitrogenase/hydrogenase enzyme system. Anaerobic fermentation would be a feasible hydrogen evolution route since there is a worldwide surplus of fermentable biowastes such as agricultural lignocellulose, domestic and kitchen wastes, etc. Biomass is one of the most promising and stable resources anticipated to produce different types of biofuels such as biodiesel, bioethanol, biogas, biohydrogen and biobutanol etc. [41]. Using different biomasses carbon sources, Danial et al. studied fermentative hydrogen production by purple non sulfur bacteria [4, 15–17, 32] such as orange peel [60], municipal solid waste [22] and paperboard mill sludge [26, 88]. Starch enriched *Chlamydomonas* biomass or different excreted end products derived from starch mobilization can be used by purple non-sulfur photosynthetic or heterotrophic bacteria to produce H_2_ [23, 24]. Algal biomass, in particular, is one of the most promising bioenergies which can be further processed to various biofuels e.g. fermentation by bacteria into hydrogen, due to its availability in massive and cheap amounts; the environmental role of algae to consume (photosynthesize) atmospheric CO_2_ during their growth should be also considered. The production of biohydrogen from microalgae through dark fermentation has received increasing attention in recent years [43]. The production of hydrogen from algal biomass by bacterial fermentation is under continuous consideration to find out feasible cost benefit ratio, e,g., from *Chlorella* [1, 27]. The production of hydrogen from algal biomass through thermochemical route was also assessed [40]. For algal biomass, the optimum carbon–nitrogen ratio of fermentation is usually considered to be 20–30 [58].

Microalgal cell size is also pivotal for biomass productivity and intracellular metabolism. Planktonic algae growth requires the uptake of nutrients from the medium to form new cells [52]. Small cells have a large surface area to volume ratio, which facilitates rapid assimilation of nutrients [62]. Under the condition of environmental stress, the volume of microalgae cells will increase.

In summary of this study, *Chlorococcum humicola* grew at the highest rate and had the highest dry weight at C/N ratios of 1:10 and 1:1. These were associated with a higher oil content. Moreover, *Bacillus coagulans* produced hydrogen by using the algal cake remaining material from the lipid extraction process as biomass.

## Conclusions

Photosynthesizing carbonate, which exists in massive amounts on earth, may imply positive interaction with global carbon emission and cycle. In this hypothesis, carbonate plays a dual role of source/ sink, from which CO_2_ is photosynthesized and at which it is fixed. Carbonate/ nitrate combinations, at certain ratios of 1:10 and 1:1, represented the optimum concentrations for growth of *Chlorococcum humicola*, oils and hydrogen evolution; these ratios resulted in the highest dry mass along with up to 30% of the dry mass as oils. Fatty acids exhibited different profiles dependent on carbonate/ nitrate combinations.

## Data Availability

No datasets were generated or analysed during the current study.
